# Pharmacokinetic and Pharmacodynamic Considerations for the Use of Monoclonal Antibodies in the Treatment of Bacterial Infections

**DOI:** 10.3390/antib7010005

**Published:** 2018-01-04

**Authors:** Shun Xin Wang-Lin, Joseph P. Balthasar

**Affiliations:** Department of Pharmaceutical Sciences, University at Buffalo, State University of New York, Buffalo, NY 14214, USA; sl256@buffalo.edu

**Keywords:** bacterial infections, monoclonal antibodies, pharmacokinetics, pharmacodynamics

## Abstract

Antibiotic-resistant bacterial pathogens are increasingly implicated in hospital- and community-acquired infections. Recent advances in monoclonal antibody (mAb) production and engineering have led to renewed interest in the development of antibody-based therapies for treatment of drug-resistant bacterial infections. Currently, there are three antibacterial mAb products approved by the Food and Drug Administration (FDA) and at least nine mAbs are in clinical trials. Antibacterial mAbs are typically developed to kill bacteria or to attenuate bacterial pathological activity through neutralization of bacterial toxins and virulence factors. Antibodies exhibit distinct pharmacological mechanisms from traditional antimicrobials and, hence, cross-resistance between small molecule antimicrobials and antibacterial mAbs is unlikely. Additionally, the long biological half-lives typically found for mAbs may allow convenient dosing and vaccine-like prophylaxis from infection. However, the high affinity of mAbs and the involvement of the host immune system in their pharmacological actions may lead to complex and nonlinear pharmacokinetics and pharmacodynamics. In this review, we summarize the pharmacokinetics and pharmacodynamics of the FDA-approved antibacterial mAbs and those are currently in clinical trials. Challenges in the development of antibacterial mAbs are also discussed.

## 1. Introduction

The clinical application of antibodies for the treatment of infectious diseases was first introduced in the form of serum therapy in early 1890s by Emil von Behring and Shibasaburo Kitasato [1]. Serum therapy was then widely applied to treat infections caused by several bacterial pathogens, including *Corynebacterium diphtheria*, *Streptococcus pneumoniae*, *Neisseria meningitides*, *Haemophilus influenzae*, Group A *Streptococcus*, and *Clostridium tetani* [2]. Although serum therapy became the standard-of-care for several infectious diseases in the pre-antibiotic era, treatment with polyclonal antisera poses several drawbacks, including “serum sickness” or immune complex hypersensitivity that can occur in 10–50% patients, lot-to-lot variation in efficacy, low content of specific antibodies, and potential hazards in the transmission of infectious diseases [2,3,4,5]. In 1937, the discovery of sulfonamides led to a boom in the development of antimicrobial chemotherapy [6], and the significant advantages associated with antimicrobials, such as less toxicity and cost, higher efficacy, and broad spectrum activity, resulted in the abandonment of serum therapy. Drug resistance, which has been a concern from the onset of antibacterial chemotherapy, has become a major clinical problem within the past few decades. It has been suggested that broad spectrum antimicrobial activity contributes to the widespread development of resistant strains, and specific mechanisms of resistance can either exist before, or emerge rapidly after, the clinical launch of new antibiotics [7]. In 2004/5, pan-drug resistant strains (i.e., resistant to all antimicrobials currently approved by FDA) of *Acinetobacter* and *Pseudomonas* were identified [7]. Unfortunately, as drug resistance has been increasing, the rate of approval of new antibiotics has been decreasing. The number of new antibacterial drugs approved has decreased from an average of five per year in the 1980s to less than one per year in the 2000s [7]. 

The discovery of hybridoma technology in 1975 and recent advances in monoclonal antibody (mAb) engineering, which make production of unlimited amount of human mAbs possible [8,9], have renewed interest in the development of antibacterial antibody therapies. Monoclonal antibodies are widely used to treat immune deficiencies, cancers, multiple sclerosis, rheumatoid arthritis, and psoriasis, but the application of mAbs for bacterial infections has progressed slowly. Currently, there are only three mAbs approved by the FDA for use in the treatment of bacterial infections (Table 1). All three mAbs are indicated as adjuvant therapies to antibiotics and do not have bactericidal activity. They are directed against bacterial exotoxins and protect host cells from toxin-mediated cytotoxicity through neutralization of exotoxin activities. As of December 2017, there are nine mAb products in clinical trials (Table 2). Of these, six are ‘naked’ mAbs, two are mAb cocktails containing two mAbs that bind to different targets (ASN100 and Shigamab), and one is an antibody-antibiotic conjugate (DSTA4637S) that kills intracellular bacteria through the intracellular delivery of a potent antibiotic. Of note, five of the nine mAb products in clinical testing bind to the bacterial cell surface and have shown bactericidal activity in preclinical studies (DSTA4637S, 514G3, MEDI3902, Aerumab, and Aerucin); the other four target exotoxins and protect against infections via toxin neutralization (MEDI4893, ASN100, Salvecin, and Shigamab). 

In this review, we discuss important considerations of mAb-based therapies for the treatment of bacterial infections, including unique challenges, pharmacokinetic (PK) properties, and pharmacodynamic (PD) mechanisms of action. The PK/PD characteristics of FDA-approved antibacterial mAbs and those in clinical trial are also summarized.

## 2. Pharmacokinetic Considerations

All FDA-approved antibacterial mAbs and the majority of those in clinical trials are of the immune gamma globulin (IgG) isotype. IgG is the predominant immunoglobulin isotype, comprising approximately 80% of immunoglobulin in human serum. An intact IgG has a molecular weight of ~150 kDa, with two antigen binding domains and a highly conserved crystallizable region (Fc) that is responsible for binding to Fc gamma receptors (FcγRs) on immune cells and activating Fc-mediated effector functions. IgG typically exhibits linear pharmacokinetics (i.e., area under the drug concentration-time curve (AUC) is directly proportional to the dose) in healthy human subjects, with small volumes of distribution (3–9 L), relatively slow clearance (8–12 mL/h), and long half-lives (20–25 days) [10]. The long biological persistence of IgG is partially attributed to Brambell receptor (FcRn) mediated salvage of IgG from lysosomal catabolism [11]. In contrast to the pharmacokinetics of pooled endogenous IgG, therapeutic mAbs often demonstrate nonlinear PK (i.e., AUC is not proportional to the dose), depending on the total body load of the pharmacological target (i.e., the quantity of bacteria, in the case of antimicrobial mAb), the accessibility of the targets, mAb-target affinity, and mAb doses. Key PK considerations for antibacterial mAbs are summarized below, including a discussion of determinants of target mediated drug disposition (TMDD) and mAb distribution in infected organs. 

### 2.1. Target Mediated Drug Disposition

Target mediated drug disposition describes the phenomenon where binding of a high affinity drug to its pharmacological target affects the PK characteristics of the drug (i.e., kinetics of distribution and clearance) [12]. At relatively low doses (compared to the amount of target), high affinity mAb-target binding results in drug accumulation at the sites of action (i.e., target-expressing tissue or site of infection), which may lead to a large apparent volume of distribution of the mAb. With increased doses and increased mAb concentrations, the target sites become increasingly saturated, which may decrease tissue to plasma mAb concentration ratios, decreasing the apparent volume of distribution. Additionally, mAb-target binding may trigger receptor-mediated endocytosis, in which mAb-target complexes are engulfed and degraded in lysosomes. This target-mediated elimination accelerates the clearance of mAbs and thereby shortens their biological persistence and half-life. Due to the effect of the drug in enhancing the elimination of the target, the volume of distribution and clearance of the drug may decrease during the course of repeated dosing.

All marketed antibacterial mAbs and those in clinical trials were developed to kill bacteria or attenuate bacterial pathological activity via antibody-mediated effector functions or toxin neutralization (Table 1 and Table 2). Antibody-toxin and antibody-bacteria complexes may be cleared by phagocytic cells through Fc engagement with FcγRs, and via subsequent endocytosis and catabolism in phagolysosomes. Thus, antibacterial mAbs may be expected to exhibit TMDD characteristics. However, the pharmacokinetics of antibacterial mAbs have not been well evaluated to date. Obiltoxaximab, a chimeric IgG1 targeting the protective antigen of *Bacillus anthracis*, showed ~2-fold faster clearances in rabbits and monkeys challenged with *B. anthracis* spores compared to those in non-infected animals [13]. Similarly, a mAb directed against *Staphylococcus aureus* also demonstrated significantly increased clearance (12.1–15.8 mL/day/kg) and decreased half-life (3.74–5.28 days) in *S. aureus* infected mice compared to those in non-infected mice (4.69–5.19 mL/day/kg and 16.4–18.0 days, respectively) [14]. In contrast, pulmonary infection with *Acinetobacter baumannii* did not impact the PK of an anti-K2 capsule mAb in mouse blood, although a substantial accumulation of mAb (5.64–36.1 fold higher amount) was observed in tissues (i.e., lung, liver, and spleen) with high bacterial load compared to values found in non-infected mice [15]. Further investigations are needed, as TMDD results in nonlinear PK, which impacts the design of efficacious dosing regimens, and contributes to potential intra- and inter-patient PK variability (e.g., due to differences in bacterial burden and immune status). 

### 2.2. Distribution of mAbs in Infected Tissues

The tissue disposition of anti-bacterial mAbs may be complex, involving extravasation of mAb molecules from blood to tissue interstitial fluids, diffusion of the molecule in the interstitial fluids to bacterial targets, binding to bacterial targets, and elimination of mAbs from tissue via convective drainage through the lymphatics and via catabolism. Extravasation of IgG antibodies is typically thought to be governed by both diffusion and convection (i.e., bulk movement of molecules through paracellular pores in the vascular endothelium), but convection has been estimated to contribute more than 98% of the total transport [16,17,18]. Bacterial invasion and dissemination normally accompany disruption of the vascular endothelial integrity due to bacterial toxin-mediated cytotoxicity. This vascular damage may lead to increased antibody extravasation within infected tissues. Once bacterial cells seed in the tissue, rapid bacterial growth and release of exotoxins stimulate immune responses, including recruitment of effector cells (i.e., lymphocytes, polymorphonuclear leukocytes, and phagocytes) and massive release of cytokines and chemokines. These reactions on one hand result in increased fluid infiltration and increased vascular permeability that facilitate mAb extravasation; but, on the other hand, build up fluid pressure within tissue that hampers antibody distribution (by decreasing the hydrostatic pressure gradient driving convective transport of mAb from blood to tissue interstitial fluid) [19,20]. In addition, antibody-dependent phagocytosis accelerates the elimination of mAbs (i.e., target-mediated elimination) in the tissue. Bacterial infections in visceral organs may also result in formation of abscesses, which enclose bacteria by pseudocapsules and protect them from immune cells and mAbs. Furthermore, many pathogenic bacteria generate biofilms that are comparatively inaccessible to antibodies, immune cells, and even small molecule antibiotics. Another concern is that biofilms may mediate a near continuous release of virulence factors, such as exopolysaccharides of *Staphylococcus epidermis*, that act as decoys to reduce mAb molecules reaching the bacteria [21]. Therefore, formation of both abscesses and biofilms create barriers to mAb distribution, and hence adversely affect the antibody-mediated clearance of bacteria. 

## 3. Pharmacodynamic Mechanisms of Action

Antibacterial mAbs have been developed against a variety of bacterial cell surface targets (i.e., proteins and polysaccharides) and soluble exotoxins (Table 1 and Table 2). The potential pharmacodynamic mechanism of action depends on the nature of the target, its role in bacterial pathogenesis, and mAb isotype and structure (i.e., intact IgG mAb or IgG fragments, immunoconjugates, bispecific antibodies, etc.). Anti-exotoxin mAbs typically attenuate bacterial pathological activity via neutralization of exotoxins. Monoclonal antibodies targeting bacterial surface epitopes are expected to increase bacterial clearance through enhancing antibody-dependent phagocytosis, and/or complement-mediated bactericidal activity, or via immune system-independent bacterial killing. In addition, there has been increasing interest in the development of immunoconjugates and immunomodulatory mAbs that either carry potent antimicrobials or stimulate exhausted immune effector functions to augment bactericidal activity. 

### 3.1. Toxin Neutralization

Antibacterial mAbs that act through the mechanism of neutralization are typically directed against exotoxins. mAb binding to soluble exotoxins leads to the formation of antibody-toxin complexes, which are primarily cleared by the reticuloendothelial system. All three marketed antibacterial mAbs achieve effects via toxin neutralization (Table 1). The efficacy of neutralizing mAbs has been shown to be directly correlated with mAb binding affinity. Anti-protective antigen (PA) mAb with higher binding affinity showed superior protection against anthrax lethal toxin challenge in macrophage cytotoxicity assays and in a rat infection model, when compared to mAbs with relatively low affinities [22]. Additionally, antibody-FcγR engagement was found to be required for anti-PA mAb neutralization activity, where mAb-mediated protection against anthrax infection was only shown in wild-type mice but not FcγR-deficient mice [23]. However, FcγR engagement may not be required for all anti-toxin mAb. For example, MAb166, an anti-PcrV (type III secretion injectisome) antibody, blocks the delivery of *Pseudomonas aeruginosa* type III toxins to host cells [24]. A single dose of 10 µg of MAb166 Fab fragments, which lack an Fc domain, was able to confer similar protection (≥80% survival) as intact MAb166 against clinical isolates of *P. aeruginosa* in a mouse pneumonia infection model [24,25]. 

### 3.2. Opsonophagocytosis 

Opsonophagocytosis has been considered as one of the key bactericidal mechanisms of the innate immune system. Antibody-mediated opsonophagocytosis involves antibody binding to bacterial surface antigens, followed by the engagement of FcγRs on the surface of professional phagocytes (i.e., monocytes/macrophages, neutrophils and dendritic cells), which in turn trigger actin-myosin driven endocytosis of antibody-bacteria complexes [26]. Phagosome vacuoles fuse with lysosomes, which leads to formation of phagolysosomes where bacteria are catabolized. Antibody-dependent phagocytosis is readily activated at the presence of phagocytes and antibody-bacteria complexes. It has been estimated that a surface density of only 5.33–26.7 antibodies/µm^2^ (i.e., IgG density on the surface of targeted particles) is required to trigger antibody-dependent phagocytosis [27]. In one example of the significance of opsonophagocytosis for mAb treatment of bacterial infection, Russo et al. developed a mAb 13D6 against K1 capsular polysaccharide of *Acinetobacter baumannii*, which showed potent inhibitory effects on bacterial growth in a rat soft tissue infection model. Antibody-dependent phagocytic killing has been found to be the primary bactericidal mechanism in this study [28]. 

### 3.3. Complement-Dependent Cytotoxicity

Antibody-dependent (i.e., classical) complement activation is another important bactericidal mechanism of the innate immune system. Binding of antibodies on the bacterial surface enhances the recruitment and binding of soluble complement factors, including C1q, to the Fc domain of the mAb, which leads to the activation of the complement cascade (i.e., complement fixation), formation of the membrane attack complex, thus leading to bacterial killing. Activation of the antibody-dependent pathway requires interaction of C1q with at least two IgG molecules [29]. Based on the molecular size of C1q, it is estimated that the surface density of IgG must be such that IgG molecules are separated by no greater than ~40 nm to fix C1q [30]. In contrast, IgM is much more efficient in complement activation, as a single IgM molecule is able to fix C1q [29]. An anti-keratin antibody IgM (3B4) directed against Methicillin-resistant *Staphylococcus aureus* (MRSA) was generated by An et al. with strong binding to MRSA and mannose-binding lectin (MBL), which in turn activated the classical and MBL complement pathways and led to potent bactericidal activity [31]. Passive immunization with 3B4 significantly decreased bacterial burden in organs and improved animal survival in a mouse bacteremia model [31].

### 3.4. Direct Bactericidal mAbs

In addition to the mechanisms of action discussed above, antibacterial mAbs showing direct bactericidal activity have been occasionally identified. Binding of these mAbs may trigger lysis of bacterial cells directly (i.e., without requirement for fixation of complement of engagement of other components of the host immune system). For instance, LaRocca et al. developed an IgG1 mAb (CB2) that targets outer surface protein B (OspB) of *Borrelia burgdorferi* for the treatment of Lyme disease [32]. CB2 exhibited complement-independent pore forming when bound to *B. burgdorferi*, which resulted in osmotic lysis of bacterial cells. However, this bactericidal effect was not transferable to *Escherichia coli* expressing recombinant OspB, suggesting a unique interaction between CB2 and *B. burgdorferi* [32]. The underlying mechanism of action is unclear, but it was found to be correlated with cholesterol glycolipids in the *B. burgdorferi* outer membrane that exist as temperature-sensitive lipid raft-like microdomains [33].

### 3.5. Immunoconjugates

The use of mAbs to deliver highly potent payloads has been successfully applied in cancer treatment [34]. This strategy, on one hand, does not require the mAb itself to be protective; on the other hand, it increases the half-life and specificity of the payload and, hence, decreases off-target toxicity. However, application of this approach to bacterial infection is still in its infancy. In 2015, Lehar and colleagues for the first time adapted the immunoconjugate strategy to antimicrobials and developed a novel THIOMAB™ (Genentech, South San Francisco, CA, USA) antibody antibiotic conjugate (AAC) against *Staphylococcus aureus* [35]. The antibody module opsonizes *S. aureus* and mediates uptake into phagolysosomes, where a potent antibiotic payload is released, allowing efficient killing of intracellular bacteria [35]. This AAC strategy demonstrated promising bactericidal activity against vancomycin-resistant *S. aureus*, and it was especially efficacious for bacteria with an intracellular life cycle [36]. Antibody-antibiotic conjugates may be expected to demonstrate favorable pharmacokinetics (i.e., long half-lives), and decreased off-site toxicity. For example, the AAC strategy ameliorated antibiotic-mediated disruption to the normal flora, and may decrease selective pressure that enhances the development of cross resistance, due to the specificity provided by the antibody carrier. The advantages provided by antibody conjugation may allow for reconsideration of antimicrobials that failed in development due to unfavorable PK or toxicity. 

Radioimmunoconjuates, which link radionuclides to mAbs, may allow targeted delivery of bactericidal radiation to bacteria. As a proof-of-principle, Dadachova et al. developed a radioimmunoconjugate with Bismuth-213 linked to a mAb (D11) targeting the pneumococcal capsular polysaccharide [37]. Administration of ^213^Bi-D11 showed dose-dependent bacterial killing in vitro and in a mouse bacteremia model, without detectable hematological toxicity [37]. In addition, radioimmunotherapy has also shown to confer protection against human immunodeficiency virus (HIV) in severe combined immunodeficiency (SCID) mice, and to selectively kill HIV-infected human T cells and human peripheral blood mononuclear cells in vitro [38]. These data suggest that radioimmunoconjugates may provide protection in immunocompromised patients and may be efficacious against infected host cells that express bacterial antigens on cell surfaces, which could be a novel approach to clear latent intracellular bacteria.

### 3.6. Immunomodulatory mAbs

Immunomodulatory mAbs, such as T-cell engaging antibodies and antibodies targeting programmed cell death protein 1 (PD-1) or cytotoxic T lymphocyte-associated protein 4 (CRLA-4), have gained great success in the treatment of cancer. However, their application in bacterial infections has not been well explored. Akin to cancer, chronic exposure of antigens to T-cells during persistent infections leads to cellular exhaustion of effector functions [39]. Thus, immunomodulatory mAbs theoretically may aid in the clearance of bacteria through stimulating the host immune system. Recently, evidence from the literature supports the potential benefit of anti-PD-1 mAb for the treatment of tuberculosis (TB) infection. PD-1 and its ligands (PD-L1 and PD-L2) were found to be significantly decreased in CD4^+^ and CD8^+^ T cells in TB patients after standard-of-care therapy [40]. Treatment with anti-PD-1 mAb has been shown to restore cytokine secretion and antigen responsiveness of T cells isolated from TB patients ex vivo [41].

## 4. Challenges in the Development of mAbs for the Treatment of Bacterial Infections

Development of antibacterial mAbs has been progressing relatively slowly. Only three antibacterial mAbs have been marketed in the United States (Table 1), and nine mAb products are in Phase 1–2 clinical trials (Table 2). Although modest success in this area may be partly due to a real or perceived lack of economic incentive to the pharmaceutical industry, limited development of antibacterial mAbs may also relate to a host of scientific challenges. Some of the complexities include difficulties in the selection of accessible and conserved bacterial targets, risk for antibody-dependent enhancement of bacterial infection, and various bacterial countermeasures against antibodies. 

### 4.1. Difficulties in Selection of Bacterial Targets

Antibacterial mAbs have been primarily developed to target either bacterial cell surface targets or secreted exotoxins. Although anti-exotoxin antibodies have been successful, these mAbs only attenuate bacterial pathological activity though neutralization of toxins, and are not expected to provide bactericidal activities. Anti-exotoxin mAbs, therefore, are typically indicated for prophylaxis or as adjunctive therapies to antibiotics. Antibacterial mAbs directed against bacterial surface epitopes have primarily been developed for binding to outer membrane proteins (OMPs) or exopolysaccharides (i.e., capsules or O-antigens of lipopolysaccharides) as shown in Table 2. OMPs are attractive therapeutic targets for vaccination and passive immunization due to the high conservation of OMPs among clinical isolates. Outer membrane protein A (OmpA)-like proteins, for instance, are conserved across all sequenced clinical isolates with high protein homology in many Gram-negative bacteria such as *Escherichia coli*, *Pseudomonas aeruginosa*, and *Acinetobacter baumannii* [42,43]. However, a concern for mAbs targeting OMPs are reports that exopolysaccharides mask these conserved targets, impede mAb binding, and hinder opsonization [44,45,46,47,48]. In contrast, exopolysaccharides are readily accessible to antibody binding. Several of the mAbs in development are directed against exopolysaccharides, such as DSTA4637S, MEDI3902, Aerumab, and Aerucin (Table 2). However, exopolysaccharide epitopes are not typically conserved. Large numbers of capsular serotypes have been identified for many bacterial pathogens. For example, at least 18 and 90 capsular serotypes have been described for *Staphylococcus aureus* and *Streptococcus pneumoniae*, respectively [49,50]. A single mAb thus may only provide protection against a specific capsular serotype. For instance, Aerumab binds only to serotype O11 of *P. aeruginosa* (Table 2) and, therefore, mAb cocktails that recognize different serotypes may be required to confer meaningful antibacterial efficacy. In addition, exopolysaccharides may shed from bacterial cells and act as decoys, which may reduce the amount of unbound antibody reaching the bacterial surface. Capsular polysaccharides shed from *Klebsiella pneumoniae*, *Streptococcus pneumoniae*, and *P. aeruginosa* have been shown to increase the resistance to antimicrobial peptides (e.g., polymyxin B and neutrophil α-defensin 1) of an unencapsulated strains (i.e., 3-fold increase in minimum inhibitory concentrations). Incubation with these antimicrobial peptides also stimulated the release of capsular polysaccharide [51]. Shed capsular polysaccharides from *A. baumannii* appear to neutralize free anti-capsule mAb molecules and may contribute to the lack of efficacy of mAb treatment in an *A. baumannii* mouse pneumonia infection model [15]. 

### 4.2. Antibody-Dependent Enhancement of Infection

Although the role of immunoglobulin in host defense against invading microorganisms via activation of effector cells and complement is undisputed, accumulating evidence supports that, in some instances, antibody augments microorganism infection via assisting in their colonization and invasion to host cells. Antibody-dependent enhancement (ADE) of infection was first discovered in Murray Valley encephalitis virus by Hawkes [52], and it was found to also have relevance for other viruses, such as Dengue virus, human immunodeficiency virus, Ebola virus, and Zika virus [53,54,55,56,57]. ADE of viral infection is mainly due to the intracellular viability of viruses, where binding with mAb facilitates viral adherence and entry to host cells through interaction with Fc receptors or complement receptors [58,59]. ADE of bacterial infection has been reported less frequently; however, striking mechanisms have been identified. IgA1-dependent enhancement of pneumococcal adherence to Detroit 562 pharyngeal epithelial cells found by Weiser et al. is a particular example. IgA1 protease secreted by *Streptococcus pneumoniae* cleave the anti-capsule IgA1 mAb that is bound on the bacterial exopolysaccharide. Positive charges on the IgA1 Fab fragments neutralize the negative charges on the polysaccharide, which unmasks phosphorylcholine underneath the capsular polysaccharide, thus enhancing *S. pneumoniae* binding to epithelial cells [60]. The increase in *S. pneumoniae* adherence was found to be directly correlated with the isoelectric points of the IgA1 Fab fragments [60]. Recently, antibodies obtained from persons with latent tuberculosis were found to be protective, whereas antibodies obtained from those with active tuberculosis promote *Mycobacterium tuberculosis* infection of human lung epithelial cells and promote bacterial replication in macrophages [61]. Additionally, the activity of anti-*M. tuberculosis* mAbs (protective vs. non-protective) was shown to be correlated with both antibody isotype and glycosylation patterns [62,63]. Monoclonal antibodies directed against capsule epitopes of *Acinetobacter baumannii*, one of the three top priority pathogens listed by World Health Organization, also demonstrated ADE of infection. Our laboratory had shown that an anti-capsule mAb IgG3 enhances *A. baumannii* adherence/invasion to macrophages and human lung epithelial cells through IgG engagement of FcγRs, and this ADE of infection leads to a significant increase of animal mortality in a mouse pneumonia infection model [64].

### 4.3. Countermeasures against Antibacterial mAbs

Although antibacterial mAbs exploit pharmacological mechanisms that are distinct from those of antimicrobials and hence cross-resistance with small-molecule antibiotics is unlikely, host immunoglobulin has applied “selection pressure” to bacteria for millennia, which has led to the evolution of a variety of bacterial defense mechanisms. Countermeasures against host immunoglobulin may, in many cases, provide defense against therapeutic monoclonal antibacterial antibodies. Antibody neutralizing proteins, for instance, protein A of *S. aureus* and protein G of *Streptococcus*, are membrane proteins that bind antibody Fc domain and thus impede opsonophagocytosis and complement activation [65,66,67]. Additionally, binding of serum IgG via the Fc region decorates the bacterial surface, decreasing bacterial recognition by the immune system. Many bacteria also secrete proteinases that degrade antibodies and therefore inactivate antibody effector functions. *Streptococcus pyogenes*, for example, secrete IdeS (Immunoglobulin G-degrading enzyme of *S. pyogenes*) that specifically cleaves the γ-chain of human IgG in the hinge region, and SpeB (*Streptococcal* erythrogenic toxin B) that has a broad immunoglobulin-degrading activity toward IgG, IgA, IgM, IgD, and IgE [68,69]. Following IgG cleavage, the resultant antibody fragments (e.g., Fab or F(ab’)_2_) may compete for binding with intact antibodies and further impede antibody-mediated bactericidal activities [70]. Similar proteinases are also found in *S. aureus*, *P. aeruginosa*, *Streptococcus pneumoniae*, and *Haemophilus influenzae* [71,72,73,74]. Antibody-based therapies that rely on antibody effector functions are likely to be affected by these antibody neutralizing proteins and proteinases. Additionally, some bacteria can survive and replicate inside phagocytes, which then turn into “Trojan horses” that contribute to the systemic dissemination of bacteria and recurrence of infection. *M. tuberculosis*, for instance, inhibits fusion of lysosomes with phagosomes in macrophages, which protects bacteria from killing mediated by lysosomal constituents [75]. *Rickettsia prowazekii* escapes from phagosome vacuoles before the phagosome-lysosome fusion, likely via phospholipase-mediated dissolution of phagosome membrane [76]. *S. aureus* is resistant to killing by phagolysosomal catabolism through neutralization of toxic oxygen radicals by released catalase, superoxide dismutase, and carotenoids [36,77,78]. Thus, passive immunization that depends on opsonophagocytosis alone may be ineffective against these bacteria, and antibody-antibiotic conjugates that release potent antibiotic molecules intracellularly may be an effective strategy to kill these intracellular bacteria [35].

## 5. Currently Marketed mAbs for the Treatment of Bacterial Infections

### 5.1. Raxibacumab 

Raxibacumab (Abthrax) is an anti-protective antigen (PA) mAb that has been approved for the treatment of adult and pediatric patients with inhalational anthrax due to *Bacillus anthracis*. Raxibacumab is approved for use in combination with appropriate antibacterial drugs, and for prophylaxis of inhalational anthrax when alternative therapies are not available or are not appropriate. Raxibacumab binds free PA with a high affinity (equilibrium dissociation constant K_d_ = 2.78 nM), which inhibits engagement of PA to its cellular receptors on macrophages. The antibody impedes intracellular entry of anthrax lethal factor and edema factor, which contribute substantially to the pathogenic effects of anthrax toxin [79,80]. Raxibacumab demonstrated linear PK in the dose range of 1–40 mg/kg following single IV doses in healthy human volunteers with a half-life of 20–22 days [81,82]. Co-administration with ciprofloxacin, a standard-of-care (SoC) antibiotic for *B. anthracis* bacteremia, did not affect the PK of raxibacumab [82]. Likewise, raxibacumab did not alter the PK of ciprofloxacin. 

Raxibacumab is the first biologic product that was developed and approved under the FDA Animal Rule that may be applied when it is not ethical or feasible to conduct controlled clinical trials in humans. The effectiveness of raxibacumab for treatment of inhalational anthrax thus is based on efficacy studies in rabbits and monkeys. Treatment with raxibacumab was initiated when PA was detected in serum (28–42 h) or when body temperature was sustained above baseline for 2 h in animals after challenge with aerosolized *B. anthracis* spores. Significantly improved survival was demonstrated in infected New Zealand White (NZW) rabbits and cynomolgus macaques (44% and 64% survival, respectively), when treated with 40 mg/kg raxibacumab compared to placebo groups (0% survival) [82]. In addition, the combination of raxibacumab and levofloxacin provided significantly enhanced protection (82% survival) compared to the antibiotic alone (65% survival) in *B. anthracis* challenged NZW rabbits [83]. Based on the observed and simulated systemic exposure of raxibacumab in animals versus humans, a single intravenous dose of 40 mg/kg was suggested to provide protection in humans. 

### 5.2. Obiltoxaximab

Obiltoxaximab (Anthim) is also an anti-PA mAb that was approved for the same indication and usage as raxibacumab. However, premedication with diphenhydramine is recommended and close monitoring of individuals who receive obiltoxaximab is also required due to common adverse reactions including hypersensitivity (10.6%, 34/320 healthy subjects) and anaphylaxis (0.9% cases) observed in Phase 1 clinical trials [84]. Obiltoxaximab (K_d_ = 0.33 nM) protects against anthrax toxin through inhibition of PA binding to cellular receptors on host cells [85]. Obiltoxaximab demonstrated linear PK in dose range of 4–16 mg/kg following single IV administration in healthy humans. Although obiltoxaximab PK has not been studied in infected patients [84], infection of NZW rabbits and cynomolgus monkeys with *B. anthracis* led to significantly faster clearance (17.0 mL/day/kg and 8.6 mL/day/kg) compared to values observed in non-infected animals (8.7 and 4.2 mL/day/kg, respectively) [13]. These data are suggestive of target-mediated elimination upon binding of obiltoxaximab to PA; however, this hypothesis requires further investigation. The estimated half-life and volume of distribution of obiltoxaximab in healthy volunteers was 17–23 days and 6.3–7.5 L, respectively [84]. Low titers (1:20–1:320) of anti-obiltoxaximab antibodies were detected in eight subjects (2.5%) during phase 1 studies, but alterations in PK and toxicity profile were not observed in these individuals [84]. Further, the PK of obiltoxaximab was not affected by concomitant intravenous and oral doses of ciprofloxacin in healthy humans and vice versa [84]. 

Obiltoxaximab was also approved under the US FDA Animal Rule. Therapeutic efficacy of obiltoxaximab was assessed in animals challenged with aerosolized *B. anthracis* spores. The mAb was administered after animals exhibited clinical signs of systemic anthrax (i.e., presence of PA in serum or sustained elevation of body temperature above baseline), and intravenous obiltoxaximab at 16 mg/kg was able to significantly improve survival in NZW rabbits (62–93%) and cynomolgus macaques (31–47%) compared to 0–6% survival in placebo groups [86]. In prophylaxis studies, a single dose of obiltoxaximab (16 mg/kg) administered 24–72 h prior to *B. anthracis* infection provided full protection (i.e., 100% survival) in cynomolgus macaques versus 10% survival in control animals [13]. Furthermore, obiltoxaximab administered in combination with antibiotics such as levofloxacin, ciprofloxacin, and doxycycline resulted in higher survival rates than the antibiotic alone in *B. anthracis* infected animals [13]. 

### 5.3. Bezlotoxumab

Bezlotoxumab (Zinplava) is a human IgG1 that has been approved for use to reduce recurrence of *Clostridium difficile* infection (CDI) in patients ≥18 years of age who are receiving antibacterial drugs for CDI and are at high risk for CDI recurrence. Bezlotoxumab binds with high affinity (K_d_ < 1 nM) to toxin B, a pivotal virulence factor of *C*. *difficile*. The mAb inhibits toxin B binding to host cells and hence prevents toxin B-mediated inactivation of Rho GTPases and downstream signaling pathways in cells [87]. The PK of bezlotoxumab was assessed in *C*. *difficile*-infected patients in Phase 3 clinical trials, with estimated mean clearance, volume of distribution, and half-life of 0.317 L/day, 7.33 L, and 19 days, respectively [88]. Recurrence of CDI (i.e., development of a new episode of *C. difficile*-associated diarrhea following clinical cure of the presenting CDI episode) was significantly lower in patients receiving 10 mg/kg bezlotoxumab with SoC (17.4% and 15.7%) than the subjects receiving placebo with SoC therapy (27.6% and 25.7%) in two Phase 3 studies [89]. However, addition of bezlotoxumab to SoC did not improve clinical cure rate in *C*. *difficile*-infected patients compared to the SoC group [89]. Thus bezlotoxumab is indicated only for prevention of recurrence of CDI, but not for treatment of CDI. 

## 6. Antibacterial mAbs in Clinical Trials

In addition to the three marketed mAb products, there are nine mAbs that are currently being investigated in clinical trials. Among the nine products listed in Table 2, five mAbs are developed against *Staphylococcus aureus*, three are targeting *Pseudomonas aeruginosa*, and one is for *Escherichia coli*. Released data from preclinical and clinical studies are summarized here to give a broad overview of the products that may be clinically available in the next few years. 

### 6.1. MEDI4893

MEDI4893 is a human IgG1 mAb that specifically binds to and neutralizes alpha-toxin (AT) of *Staphylococcus aureus* and hence inhibits AT-mediated cytotoxic activity toward host cells [90]. AT is a 33-kDa pore-forming toxin that forms heptameric pores in host cells membranes and results in cell lysis [91]. Animal studies using isogenic AT negative mutants demonstrated that AT is a key virulence factor in *S. aureus* infections including sepsis, skin and soft tissue infection, and pneumonia [91,92,93]. AT was found to be expressed in 83% clinical isolates worldwide, and 91% of the isolates encoded AT subtypes that were neutralized by MEDI4893 [90]. In an acute pneumonia infection model, MEDI4893 was shown to provide both prophylactic and therapeutic effects in immunocompetent and immunocompromised mice. Further, sub-therapeutic MEDI4893 doses administered in combination with sub-therapeutic doses of antibiotics (vancomycin or linezolid) provided significantly improved survival rates compared to monotherapies [94,95]. In a recent phase 1 clinical trial, MEDI4893 exhibited linear PK in the dose range of 225–5000 mg/subject. YTE mutations (amino acid substitutions M252Y/S254T/T256E) in Fc region of the mAb, which increase binding affinity for FcRn, contribute to its favorable clearance of 42–50 mL/day and extended half-life of 80–112 days [96]. 

### 6.2. ASN100

ASN100 is a mAb combination of two human IgG1, ASN-1 and ASN-2, which is in development for the prevention of ventilator-associated *S. aureus* pneumonia (VASP). ASN-1 targets alpha-toxin and four leukocidins including gamma hemolysins (HIgAB and HIgCB), Panton-Valentine leukocidin (LukSF or PVL), and LukED. ASN-2 binds another leukocidin LukGH (LukAB) [97,98]. Leukocidins are pore-forming toxins that typically lyse human phagocytic cells and thus play a key role in bacterial evasion of the innate immune response [99,100,101,102]. Therefore, ASN100 binds six different toxin molecules to protect against lysis of multiple human cells, including polymorphonuclear leukocytes, monocytes, macrophages, red blood cells, T cells, epithelial, and endothelial cells. Among the five leukocidins, HIgAB, HIgCB, and LukGH are highly conserved in *S. aureus* clinical isolates. LukED is expressed in 50–75% isolates, while LukSF is only present in 5–10% isolates but is correlated with more severe infections [99]. ASN-1 and ASN-2 exhibit linear serum PK over the dose range of 200–4000 mg/subject either when administered alone or simultaneously in healthy human volunteers [103]. Estimated mean clearance, volume of distribution, and half-life of ASN-1 are 0.256 L/day, 7.14 L, and 23.5 days. Values for ASN-2 are 0.186 L/day, 6.45 L, and 26.7 days [103]. 

### 6.3. DSTA4637S

DSTA4637S is a THIOMAB™ (Genentech, South San Francisco, CA, USA) antibody antibiotic conjugate (AAC) that is comprised of an anti-*S. aureus* THIOMAB™ (Genentech, South San Francisco, CA, USA) antibody and a potent antibiotic, 4-dimethylamino piperidino-hydroxybenzoxazino rifamycin (dmDNA31), linked through a protease cleavable valine-citrulline linker [35]. It has been known for more than half-century that *S. aureus* can survive inside neutrophils and turn them into “Trojan horses”, which assist in systemic bacterial dissemination and contribute to recurrence of infection following antibacterial therapy [104]. DSTA4637A (a preclinical formulation of DSTA4637S) demonstrated potent intracellular bactericidal activity against *S. aureus* both in vitro and in a mouse bacteremia model [35]. The antibody module of the AAC specifically targets the β-*O*-linked *N*-acetylglucosamine sugar modifications on cell wall teichoic acid residues of *S. aureus* and is responsible for opsonization of bacteria. Once the opsonized bacteria are taken up into phagolysosomes, proteases such as cathepsins cleave the linker and release the potent dmDNA31 antibiotic, which eradicates intracellular *S. aureus* [35]. Total concentrations of the DSTA4637A antibody (TAb) and antibody-conjugated dmDNA31 (ac-dmDNA31) were consistent with linear plasma PK over the dose range of 5–50 mg/kg in non-infected mice and 25–50 mg/kg in *S. aureus* bacteremia mice [14]. Infection with *S. aureus* had negligible impact on plasma PK of TAb and ac-dmDNA31 over the efficacious dose range of 25–50 mg/kg, with mean clearance of 4.95 vs. 6.08 mL/day/kg, volume of distribution of 94.9 vs. 119 mL/day/kg, and half-life of 14.3 vs. 13.9 days for TAb in non-infected and infected mice, respectively [14]. PK data for DSTA4637S in human subjects have not been published. 

### 6.4. Salvecin

Salvecin (AR-301) is a mAb developed as an adjunctive therapy to SoC antibiotics for ventilator-associated *S. aureus* pneumonia [105]. It binds and neutralizes alpha-toxin and hence prevents AT-mediated lysis of host cells. Phase 1/2a study results met their primary endpoints, and showed that VASP patients who received Salvecin in combination with SoC antibiotics spent shorter time under mechanical ventilation than patients treated with placebo plus antibiotics [106]. In addition, blood bacterial burden was consistently lower in Salvecin-treated patients compared to the control group [106]. 

### 6.5. 514.G3

514G3 is a human mAb targeting *Staphylococcus* Protein A (SpA), a key virulence determinant of *S. aureus* that is expressed in all clinical isolates [107]. SpA is present in the *S. aureus* cell wall envelope and is released during bacterial growth [108]. SpA binds the Fc domain of human IgG and protects *S. aureus* from antibody-dependent phagocytic killing [109,110]. Additionally, released SpA triggers B cell superantigen activity through cross-linking of B cell receptors at V_H_3 domain [111,112]. 514G3 displaces SpA-bound serum IgG on *S. aureus* surface and enhances opsonophagocytosis or other mechanisms of immune clearance of bacteria [107]. In a pilot Phase 2 study in patients hospitalized with *S. aureus* bacteremia, treatment with 40 mg/kg 514G3 led to 49% reduction in relative risk of overall incidence of serious adverse events (SAEs) and 56% relative risk reduction in *S. aureus* related SAEs compared to the placebo group [113]. More importantly, the duration of hospitalization was reduced by 33% in 514G3 treated patients compared to patients who received placebo (8.6 ± 7 days vs. 12.7 ± 9 days, respectively) [113]. 

### 6.6. MEDI3902

MEDI3902 is a bispecific mAb that targets both type III secretion injectisome PcrV anchored on bacterial cell wall and serotype-independent Psl exopolysaccharide of *Pseudomonas aeruginosa* [114]. PcrV is a critical component of the type III secretion system (T3SS) that delivers bacterial toxins and effector molecules into host cells in order to initiate infection. Psl exopolysaccharide is important for *P. aeruginosa* colonization/attachment to mammalian cells and formation of biofilms [115,116]. The majority of *P. aeruginosa* clinical isolates express Psl (89.8–91.2%) and PcrV (87.7–90.2%), and at least one of the targets were identified in 97.3–100% of isolates [114]. While binding of MEDI3902 to PcrV inhibits T3SS-mediated cytotoxicity, targeting Psl prevents *P. aeruginosa* attachment to host cells and enhances opsonophagocytic killing of bacteria. Intravenous administration of MEDI3902 (5 or 15 mg/kg) at 24 h before or 1 h after lethal *P. aeruginosa* challenge conferred 100% survival and significant reductions on tissue bacterial burdens in acute pneumonia and bacteremia animal models including mice, New Zealand rabbits, and pigs [114,117,118]. 

### 6.7. Aerumab

Aerumab (AR-101), previously known as panobacumab, is a human IgM mAb directed against the O-antigen of *P. aeruginosa* lipopolysaccharide serotype O11, which accounts for ~20% of clinical isolates. Aerumab is being developed as an adjunctive immunotherapy to SoC antibiotics for ventilator associated pneumonia caused by *P. aeruginosa* [119,120]. Binding of Aerumab to *P. aeruginosa* leads to enhanced bacterial clearance through either phagocytosis or complement-mediated bacterial killing [120]. Aerumab demonstrated linear PK over the dose range of 0.1–4 mg/kg in healthy human volunteers, with mean clearance of 0.039–0.120 L/h, volume of distribution of 4.75–5.47 L, and half-life of 70–95 h [121]. *P. aeruginosa* infection in patients did not affect the PK of Aerumab following IV doses of 1.2 mg/kg, where estimated clearance, volume of distribution, and half-life are 0.0579 L/h, 7.5 L, and 102 h, respectively [122]. Further, all 13 patients who received three doses of 1.2 mg/kg Aerumab as an adjunctive therapy given every 72 h survived, with a mean clinical resolution rate of 85% (11/13) in 8 days compared to a rate of 64% (9/14) in 18.5 days in patients who did not receive the mAb [122,123].

### 6.8. Aerucin

As indicated above, Aerumab can only recognize ~20% of *P. aeruginosa* clinical isolates. Aerucin is a second generation anti-*P. aeruginosa* mAb that binds to alginate (i.e., exopolysaccharide) in greater than 90% of clinical isolates [124]. Aerucin is also developed as an adjunctive therapy to SoC antibiotics for hospital-acquired and ventilator-associated pneumonia caused by *P. aeruginosa*, and binding of Aerucin is also expected to augment the opsonophagocytic killing and complement-dependent bactericidal activity against *P. aeruginosa* [124]. However, preclinical and clinical study results for Aerucin have not been published. 

### 6.9. Shigamab

Shiga toxin (Stx)-producing *Escherichia coli* (STEC) is the major cause of hemorrhagic colitis by infectious agents in the United States. A serious consequence of STEC infection is hemolytic uremic syndrome (HUS) that can lead to renal failure and death in 5–15% of infected children [125]. Treatments for STEC infections are currently not available, and antibiotics may increase the risk of HUS [126]. Shigamab is a combination of two chimeric mabs, cαStx1 and cαStx2, which were developed to neutralize Stx1 and Stx2, respectively. Stx1 and Stx2 are the two major types of shiga toxin that are the key virulence factors contributing to the pathogenesis of HUS [127]. Treatment with 20 mg/kg of Shigamab conferred 90% survival in mice challenged with lethal doses of Stx1 and Stx2, whereas cαStx1 or cαStx2 alone did not protect mice against infection [128]. In addition, infection with STEC strain B2F1 in mice did not affect the PK of cαStx2 at 15 mg/kg [128]. Shigamab exhibited linear PK over the dose range of 1–3 mg/kg, and cαStx1 was shown to have greater clearance (0.38 ± 0.16 mL/h/kg) and shorter half-life (190 ± 140 h) than cαStx2 (0.20 ± 0.07 mL/h/kg and 261 ± 112 h, respectively) [129]. 

## 7. Conclusions

Pathogen-specific antibacterial mAbs have become an appealing therapeutic option due to recent advances in mAb production and engineering technologies. Antibodies kill bacteria or attenuate bacterial pathological activity via various mechanisms, including opsonophagocytosis, complement-mediated bactericidal activity, antibody-dependent cellular cytotoxicity, and neutralization of bacterial toxins. These pharmacodynamic mechanisms are distinct from those of small-molecule antimicrobials and therefore, such mAbs provide an attractive therapeutic option for antimicrobial resistant strains. The high specificity of mAbs may be expected to allow less disturbance to normal flora and less selective pressure for cross-resistance. Extended half-lives of mAbs may allow less frequent dosing and long-term prophylaxis. Antibacterial mAbs may also exhibit pharmacokinetic properties such as target-mediated drug disposition due to opsonophagocytosis or formation of antibody-toxin complexes, and there is some potential for complicated tissue distribution during the course of bacterial infection. These possible complexities require further study. Though there are only three mAbs marketed for prophylaxis or treatment of bacterial infection as of today, there is promise for a more prominent future role for antibacterial mAbs in view of their many advantages over traditional antimicrobials, and in view of the positive findings from clinical investigations of several mAbs in development. 

## Figures and Tables

**Table 1:** Food and Drug Administration (FDA)-approved monoclonal antibodies (mAbs) for use in bacterial infection.

Antibody	Company	Format	Pathogen/Target	First Approved Indication	Reported Mechanism of Action	Approval Year
Raxibacumab	GlaxoSmith Kline	Human IgG1(λ)	*Bacillus anthracis*/Protective antigen	Treatment and prophylaxis of inhalational anthrax	Toxin neutralization	2012
Obiltoxaximab	Elusys	Chimeric IgG1(κ)	*Bacillus anthracis*/Protective antigen	Treatment and prophylaxis of inhalational anthrax	Toxin neutralization	2016
Bezlotoxumab	Merck & Co.	Human IgG1	*Clostridium difficile*/Enterotoxin B	Prevention of *Clostridium difficile* infection recurrence	Toxin neutralization	2016

**Table 2:** mAbs currently in clinical trials.

Antibody	Sponsor	Format	Pathogen	Target	Reported Mechanism of Action	Current Status
MEDI4893	MedImmune	Human IgG1(κ)	*Staphylococcus aureus*	Alpha toxin	Toxin neutralization	Phase 2
ASN100	Arsanis	Human IgG1(κ)	*Staphylococcus aureus*	Alpha toxin and five leukocidins	Toxin neutralization	Phase 2
DSTA4637S	Genentech	Human IgG1	*Staphylococcus aureus*	β-*O*-linked *N*-acetylglucosamine on wall teichoic acids	Antibody-antibiotic conjugate	Phase 1
Salvecin (AR-301)	Aridis	Human IgG1	*Staphylococcus aureus*	Alpha toxin	Toxin neutralization	Phase 1/2a
514G3	XBiotech	Human IgG3	*Staphylococcus aureus*	Protein A	Opsonophagocytosis	Phase 1/2
MEDI3902	MedImmune	Human bispecific IgG1	*Pseudomonas aeruginosa*	PsI and PcrV	Opsonophagocytosis; inhibition of cell attachment and cytotoxicity	Phase 2
Aerumab (AR-101)	Aridis	Human IgM(κ)	*Pseudomonas aeruginosa*	O-antigen (serotype O11)	Opsonophagocytosis; complement-mediated bacterial killing	Phase 2b
Aerucin	Aridis	Human IgG1	*Pseudomonas aeruginosa*	Alginate (surface polysaccharide)	Opsonophagocytosis; complement-mediated bacterial killing	Phase 2
Shigamab	Bellus Health	Chimeric IgG1(κ)	*Escherichia coli*	Shiga toxin 1 and 2	Toxin neutralization	Phase 2
